# Amentoflavone Ameliorates Carrageenan-Induced Pleurisy and Lung Injury by Inhibiting the NF-κB/STAT3 Pathways via Nrf2 Activation

**DOI:** 10.3389/fphar.2022.763608

**Published:** 2022-02-14

**Authors:** Tianhua Hou, Manshi Yang, Kun Yan, Xiaoye Fan, Xinxin Ci, Liping Peng

**Affiliations:** ^1^ Department of Respiratory Medicine, The First Hospital of Jilin University, Changchun, China; ^2^ Institute of Translational Medicine, The First Hospital of Jilin University, Changchun, China

**Keywords:** amentoflavone, Nrf2, carrageenan, pleurisy, lung injury

## Abstract

Many natural flavonoids can activate nuclear factor erythroid 2-related factor 2 (Nrf2), which is pivotal for alleviating various diseases related to inflammation and oxidative stress, including pleurisy. Amentoflavone (AMF), a biflavonoid extracted from many plants, has some beneficial bioactivities, especially anti-inflammatory and antioxidative activities. We aimed to investigate whether AMF protects against pleurisy and lung injury induced by carrageenan (Car) by activating Nrf2. Pleurisy was induced in wild-type (WT) and Nrf2-deficient (Nrf2^-/-^) mice. Then, pleural exudate and lung tissue were collected for biochemical analysis, H&E staining, immunocytochemistry and western blotting. Our results indicated that AMF protected against Car-induced pleurisy and lung injury. The Wright-Giemsa and H&E staining results showed that AMF alleviated inflammatory effusion and pathological injury. In addition, AMF decreased SOD and GSH depletion and MDA and MPO generation in the lung tissue of mice. AMF activated Nrf2 through keap-1 dissociation and subsequently increased heme oxygenase-1 (HO-1), NAD(P)H-quinone oxidoreductase 1 (NQO1), and γ-glutamylcysteine ligase (GCL) levels. Furthermore, AMF suppressed IL-1β and TNF-α levels and increased IL-10 levels in pleural exudate by blocking the proinflammatory NF-κB, signal transducer and activator of transcription 3 (STAT3) and extracellular signal-regulated kinase (ERK) pathways induced by Car. However, these antioxidative and anti-inflammatory effects were weakened in Nrf2^-/-^ mice. Moreover, AMF failed to suppress the NF-κB and STAT3 pathways in Nrf2^-/-^ mice. Our results demonstrated that AMF exerted anti-inflammatory and antioxidative effects in Car-induced lung injury and pleurisy in a Nrf2-dependent manner.

## Introduction

Pleurisy, which is caused by a variety of internal and external factors, such as immunological diseases, tumors and microbial infections, tends to threaten lung tissue and eventually leads to a series of respiratory diseases (Ryu et al., 2017; Leemans et al., 2018). Carrageenan (Car)-induced pleurisy is a representative experimental model of acute inflammation that is used to screen anti-inflammatory drugs. After Car exposure, local inflammation, polymorphonuclear leukocyte (PMN) infiltration and excessive reactive oxygen species (ROS) (including O_2_•^−^, H_2_O_2_, •OH, RO_2_•, RO•, ^1^O2, and O_3_) often occur in the pleural cavity, facilitating the inflammatory response and ultimately leading to lung injury (Gao et al., 2020). Therefore, oxidative stress and subsequent inflammation are the two main causes of Car-induced pleurisy and lung injury.

Fighting against oxidative stress and subsequent inflammation is essential for treating Car-induced pleurisy and lung injury (Gao et al., 2020). Nuclear factor erythroid 2-related factor 2 (Nrf2) has been found to be crucial in counteracting oxidative stress and inflammation (Ahmed et al., 2017). Nrf2 is repressed by binding with KEAP1 under quiescent conditions. However, under stress conditions, Nrf2 is released from Keap1 and then translocates into the nucleus, where it subsequently activates a variety of genes, including NAD(P)H-quinone oxidoreductase 1 (NQO-1), glutathione S-transferase (GST), heme oxygenase-1 (HO-1), and γ-glutamylcysteine ligase (GCL), to cope with stresses (Yamamoto et al., 2018). Therefore, we hypothesize that Nrf2 may be a treatment target for Car-induced pleurisy and lung injury.

Some reports have indicated that Car causes the inflammatory response by targeting various pathways, such as the NF-κB, NLRP3, mitogen-activated protein kinase (MAPK) and signal transducer and activator of transcription 3 (STAT3) pathways (Ahmad et al., 2015; Chedid et al., 2017; Gao et al., 2020). NF-κB, one of the important inflammatory pathways, is suppressed by IκB proteins in the quiescent state. When stimulated by stressors, such as ROS, cytokines and pathogen-associated molecular patterns (PAMPs), NF-κB is activated via IκB degradation by the IĸB kinase complex and promotes the transcription of inducible nitric oxide synthase (iNOS), cyclooxygenase 2 (COX-2) and some proinflammatory factors, such as IL-1β and TNF-α. Nrf2 can inhibit NF-κB or its downstream genes (Sivandzade et al., 2019), which suggests crosstalk between NF-κB and Nrf2. Additionally, the MAPK pathway, including extracellular signal-regulated kinase (ERK), c-Jun N-terminal kinase (JNK), and p38 MAPK, is responsible for the development of inflammation, boosting the expression of various inflammatory factors (Liao et al., 2019; Alam et al., 2020). Furthermore, the signal transducer and activator of transcription (STAT) family member STAT3 is activated by numerous cytokines and growth factors, such as IL-6, G-CSF, and epidermal growth factor. Serine phosphorylation at position 727 (S727) enhances transcriptional activity and is crucial for the expression of many cytokines, such as IL-6, IL-1β, CSF, and TNF-α (Hillmer et al., 2016; Wang et al., 2020). Based on domain motif interactions, STAT3 has been predicted by the NRF2-ome to be connected with Nrf2 (Turei et al., 2013). In turn, the involvement of Nrf2 in STAT3 inactivation, nuclear reduction and damaged DNA binding ability has been clarified (Gong et al., 2020).

Numerous compounds that possess antioxidative and anti-inflammatory bioactivities have been identified as related to Nrf2 and many inflammatory pathways (Lu et al., 2016). As a natural biflavonoid compound, amentoflavone (AMF) can be extracted from over 120 plants, such as Selaginellaceae, Cupressaceae, Euphorbiaceae and Podocarpaceae (Yu et al., 2017). AMF has been reported to exhibited many biological properties and effects, including anti-inflammatory, antioxidative, anticancer, antidiabetic, and antisenescence effects (Yu et al., 2017; Xiong et al., 2021). In addition, AMF has been shown to prevent sepsis-related lung injury through the Nrf2-GCLC pathway and cold stress-induced lung injury by NF-κB inhibition (Zong and Zhang, 2017; Cai et al., 2019). Moreover, AMF can suppress hepatocellular carcinoma by inhibiting the STAT3 pathway (Liu and Yu, 2020). However, the protective effects of AMF against Car-induced pleurisy and lung injury and the underlying mechanisms have not been explored. In the present study, we focused on the protective effects of AMF against Car-induced pleurisy and lung injury. Furthermore, we investigated whether the inhibitory effects of AMF on NF-κB and STAT3 were Nrf2-dependent.

## Materials and Methods

### Reagents and Chemicals

Amentoflavone (≥98% pure) was purchased from Chengdu Herbpurify Co., Ltd. (Chengdu, China), and carrageenan (Car) and dexamethasone (DEX) were obtained from Sigma Aldrich (St. Louis, MO, United States). Antibodies against MAPK, NF-κB, p-STAT3, β-actin, Histone3.1, NOX2, and NOX4 were purchased from Cell Signaling (Boston, MA, United States), and antibodies against KEAP-1, Nrf2, GCLC, GCLM, HO-1, and NQO1 were acquired from Abcam (Cambridge, MA, United States). MPO, MDA, SOD, and GSH kits were purchased from Jiancheng Bioengineering Institute of Nanjing (Nanjing, China), and mouse TNF-α, IL-10, and IL-1β enzyme-linked immunosorbent assay (ELISA) kits were obtained from BioLegend (San Diego, CA, United States). DCFH-DA and nuclear and cytoplasmic extraction reagent kits were purchased from Beyotime (China).

### Animals

Female C57BL/6 wild-type (WT) and female Nrf2^-/-^ mice (18–20 g, 6–8 weeks) were obtained from Liaoning Changsheng Technology Industrial Co., Ltd. After being maintained for 1 week in a standard manner, the animals were used for experiments. Our experiments were performed according to the International Guiding Principles for Biomedical Research Involving Animals and were approved by the Animal Use Committee of Jilin University.

### Grouping and Model Establishment

The WT mice were randomly divided into six groups (*n* = 5 per group) as follows: 1). control, 2). AMF (50 mg/kg), 3). Car (2%, 0.1 ml), 4). Car + AMF (30 mg/kg), 5). Car + AMF (50 mg/kg), and 6). Car + DEX (3 mg/kg) as a positive control group. In addition, WT mice and Nrf2^-/-^ mice were divided into four groups (*n* = 5 per group) as follows: 1) control, 2) Car (2%, 0.1 ml), 3) Car + AMF (30 mg/kg), and 4) Car + AMF (50 mg/kg). In the groups treated with Car, Car (2%) was injected into the right pleural cavity of each mouse under anesthesia to establish the pleurisy model. Control mice were injected in the same location with 0.1 ml of sterile phosphate buffer solution (PBS). AMF was dissolved in 1% DMSO (30 mg/kg or 50 mg/kg) and intragastrically administered to mice in the corresponding groups at a dose of 0.2 ml/20 g (body weight). In the positive control group, the mice were intraperitoneally injected with 0.2 ml DEX solution (3 mg/kg). After treated with AMF and DEX (separated by a time interval of 12 h), the mice were exposed to Car for 4 h. The animals were sacrificed by isoflurane inhalation afterwards.

### Pleural Exudate

The mice were euthanized and then injected with 1 ml of PBS containing heparin (5 U/ml) into the pleural cavity. Exudate that did not contain blood was collected. The volume injected (1 ml) was subtracted from the total volume collected. After centrifugation of the exudate, the cell pellets were resuspended in PBS. The total number of cells was determined with a hemocytometer, and the different types of inflammatory cells were examined by Wright-Giemsa staining. The supernatants were reserved to measure total protein concentrations and inflammatory cytokine levels.

### H&E Staining of Lung Tissues

After the samples were collected, fixed in neutral-buffered formalin and embedded in paraffin, the lung tissues were sectioned into 5 μm sections. The sections were stained with H&E and analyzed by using an Olympus BX41 microscope at a magnification of ×100. Lung injury was scored according to the presence of hemorrhage, neutrophil infiltration, edema, and hyaline membrane formation (each item was one point). The degree of lung injury was scored from 0 to 4 as follows: 0, no damage; 1) mild damage; 2) moderate damage; 3) severe damage; and 4) very severe damage.

### F4/80 Immunocytochemical Analysis of Lung Tissues

Sections were prepared as described above. After deparaffinization, the sections were hydrated and washed with Tris-buffered saline (TBS), blocked with 5% bovine serum albumin (BSA) for 1 h, incubated with F4/80 primary antibodies overnight and then with HRP-conjugated secondary antibodies, washed and mounted. The slides were analyzed with an Olympus BX41 microscope at a magnification of ×200.

### ELISA

The supernatants were collected as described above, and TNF-α, IL-1β, and IL-10 levels were measured with ELISA kits according to the manufacturer’s instructions (BioLegend, San Diego, CA, United States). The plates were blocked with capture antibodies overnight at 4°C. After sealing and incubating the plates with assay diluent A for 1 h, standards and diluted samples were added and were incubated for 2 h. Then, the plates were washed 4 times, detection antibodies were added, then the plates were incubated for 1 h. After being washed, sealed and incubated with avidin-HRP for 30 min, the plates were treated with TMB and incubated in the dark for 15 min. Finally, stop solution was added, and the absorbance at 450 and 570 nm was read with an enzyme-labeling instrument (Bio-Tek H1MFD, United States).

### ROS Measurement

ROS levels were assayed with DCFH-DA (Beyotime, China). The sedimented cells were resuspended in PBS, seeded in a 96-well plate and then incubated with DCFH-DA at 37°C for 20 min. Images were obtained by immunofluorescence microscopy. Alternatively, the cells were incubated with DCFH-DA in tubes and analyzed by flow cytometry.

### MDA, MPO, GSH, and SOD Measurement

After the lungs were collected and washed with saline, the levels of MPO, MDA, GSH, and SOD were measured with corresponding kits according to the manufacturer’s instructions (Jiancheng Bioengineering Institute of Nanjing, China).

### Nuclear and Cytosolic Protein Extraction

Cytoplasmic and nuclear proteins were isolated with an NE-PER Nuclear and Cytoplasmic Extraction Reagent Kit according to the manufacturer’s instructions (Beyotime, China). The fresh lungs were ground, and a mixture (A: B = 20:1, final PMSF concentration 1 mM) was added to the tissue. After the samples were centrifuged, the supernatants were collected. Reagent A mixed with PMSF was added to the precipitate. After the mixture was vortexed at the highest speed and bathed in ice for 15 min, reagent B was added to the mixture. The mixture was then vortexed at the highest speed and centrifuged at 4°C and 12,000 g for 5 min, and the supernatant was collected and mixed with the previous supernatant containing cytosolic protein. For precipitation, after the nucleoprotein extraction reagent was added, the mixture was vortexed for 30 min and centrifuged, and the supernatant containing nuclear protein, was collected.

### Western Blotting

Total protein was extracted from the fresh lung tissues, including exudative cells, which were collected 4 h after exposure to Car. The total protein was centrifuged at 12,000 rpm for 15 min at 4°C. The proteins were separated on SDS-polyacrylamide gels and then transferred onto polyvinylidene difluoride (PVDF) membranes. After being blocked with nonfat milk at room temperature, each membrane was incubated with primary antibodies overnight at 4°C and then with HRP-conjugated secondary antibodies for 1 h at 25°C. After each incubation, the membrane was washed three times with 0.05% PBST (1 ml of PBS + 0.5 μl of Tween). Finally, the bands were visualized with an ECL detection system, and the band intensities were analyzed by ImageJ gel analysis software.

### Statistical Analysis

All data are expressed as the means ± standard error of the mean (SEM). Differences among groups were analyzed via one-way analysis of variance (ANOVA) followed by Dunnett’s test and carried out using GraphPad Prism software version 5.0. Significance was considered at *p* < 0.05 or *p* < 0.01. All the experiments were repeated three times.

## Results

### AMF Protected Against Car-Induced Inflammatory Effusion in the Pleural Cavity and Lung Injury

**FIGURE 1:**
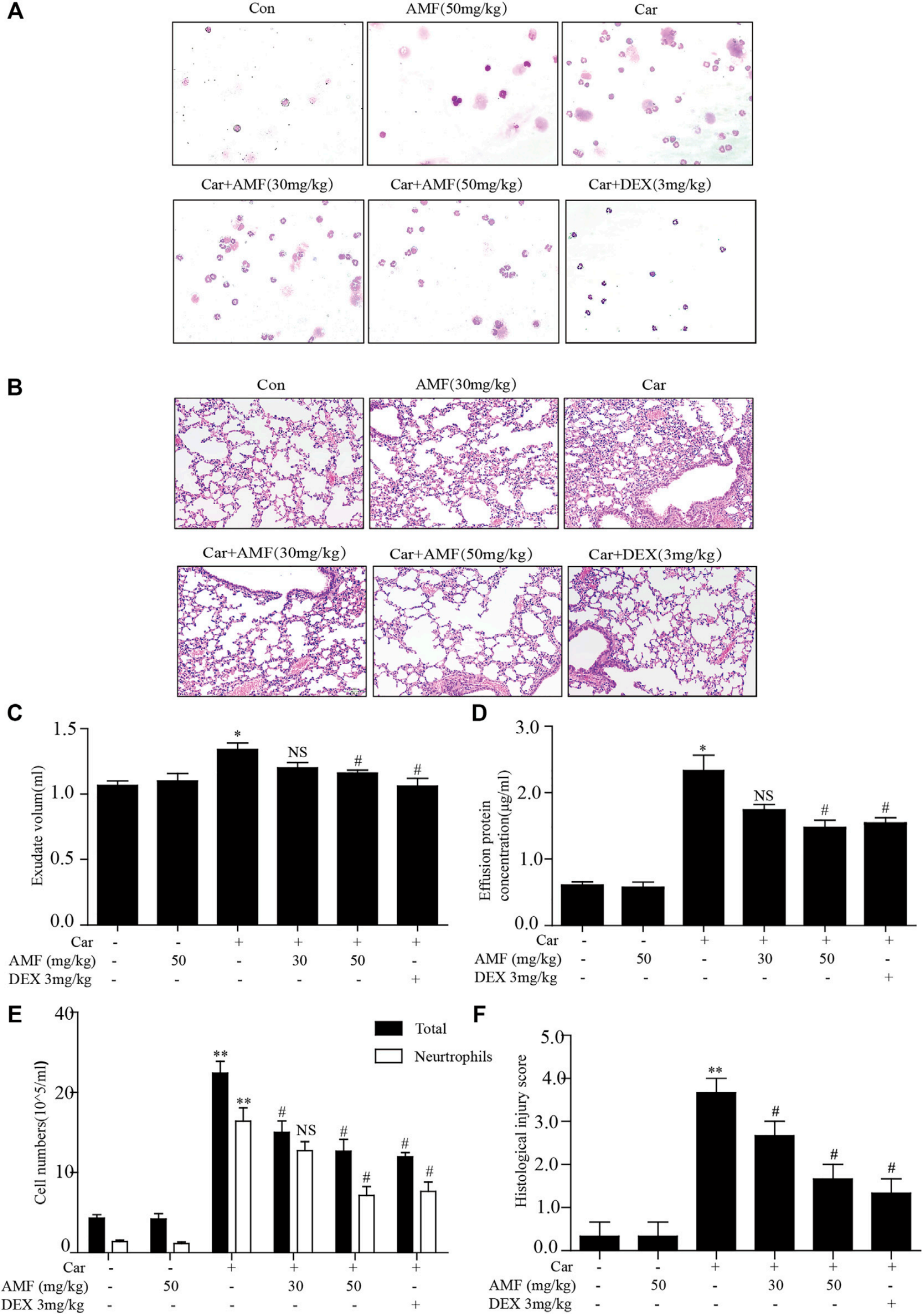
AMF protected against Car-induced inflammatory effusion and lung injury in pleurisy. **(A)** The morphologies of the different inflammatory cells were investigated by Wright-Giemsa staining (magnification ×200). **(B)** H&E staining showed the pathological changes associated with lung injury caused by Car, including inflammatory cell infiltration, interstitial thickening and alveolar hemorrhage (original magnification ×100). **(C)** Exudate volume. **(D)** The protein concentration of the pleural exudate was quantified by the BCA method. **(E)** The total number of cells and the number of polymorphonuclear neutrophils in the pleural cavity were counted with a hemocytometer. **(F)** The lung injury score; lung injury was scored according to a five-point scale as previously described. The data are expressed as the mean ± SEM. *n* = 5 per group. *p < 0.05, **p < 0.01 Car group vs. control group; #*p* < 0.05, ##*p* < 0.01, and NS *p* > 0.05 Car + AMF group vs. Car group.

First, to examine the protective effects of AMF against Car-induced pleurisy, we measured pleural effusion and pathological changes in lung tissues. DEX was used as a positive control. Wright-Giemsa staining showed that the total number of cells and the number of neutrophils in exudate were much higher in the model group exposed to Car than in the control group and that AMF treatment abrogated the Car-induced increases in total cells and neutrophils (Figures 1A,E). As shown in Figure 1B, after Car administration in the model group, pathological changes, including inflammatory cell infiltration, interstitial thickening and alveolar hemorrhage, occurred in lung tissues. However, after AMF treatment, Car-induced lung injury was significantly ameliorated. The lung injury score also indicated that AMF greatly relieved Car-induced lung injury (Figure 1F). In addition, as shown in Figures 1C,D, treatment with AMF inhibited the significant increases in effusion protein concentration and exudate volume induced by Car injection. These results indicated that AMF protected against Car-induced pleurisy and lung injury.

### AMF Alleviated the Car-Induced Inflammatory Response in Pleural Exudate and Lung Tissue

**FIGURE 2:**
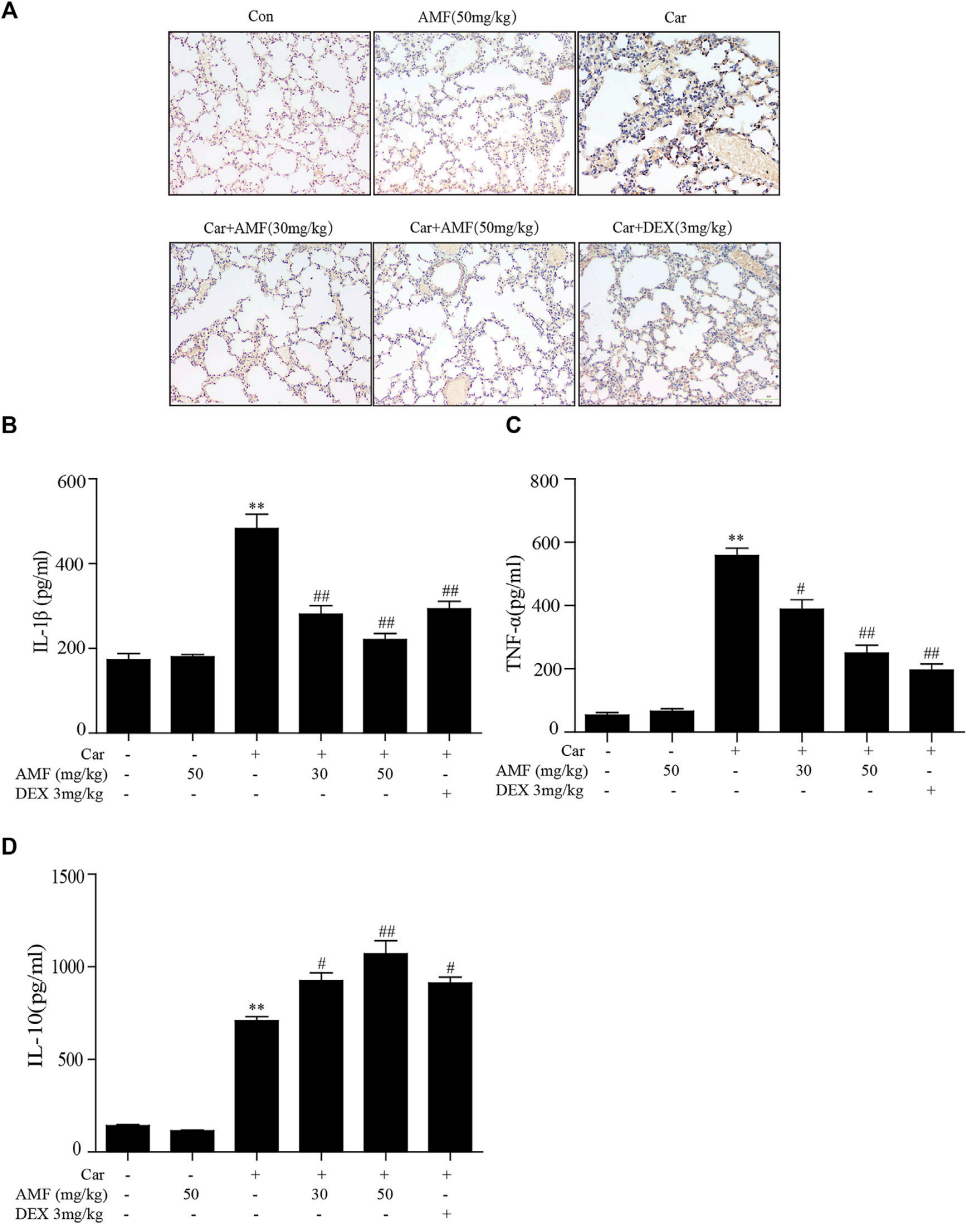
AMF alleviated the inflammatory response in pleural exudate and lung tissue. **(A)** Representative microphotographs of lung sections stained with F4/80. **(B–D)** TNF-α, IL-1β, and IL-10 levels in exudates were measured by ELISA. The data are expressed as the mean ± SEM. *n* = 5 per group. *p < 0.05 and **p < 0.01 Car group vs. control group; #*p* < 0.05 and ##*p* < 0.01 Car + AMF group vs. Car group.

To evaluate inflammatory indices, we examined related inflammatory factors and F4/80-positive cells. As shown in Figure 2A, the number of F4/80 cells sharply increased after the mice were injected with Car in the pleural cavity, whereas the number was much lower in both treatment groups. In addition, we measured the levels of related inflammatory factors in the supernatants. As shown in Figures 2B–D, the levels of IL-1β, TNF-α, and IL-10 were significantly increased by Car exposure. In the AMF treatment groups compared with the model group, the IL-1β and TNF-α levels were much lower, and the IL-10 level was higher. Our results showed the anti-inflammatory effect of AMF in Car-induced pleurisy and lung injury.

### AMF Treatment Mitigated Oxidative Stress in Car-Induced Lung Injury

**FIGURE 3:**
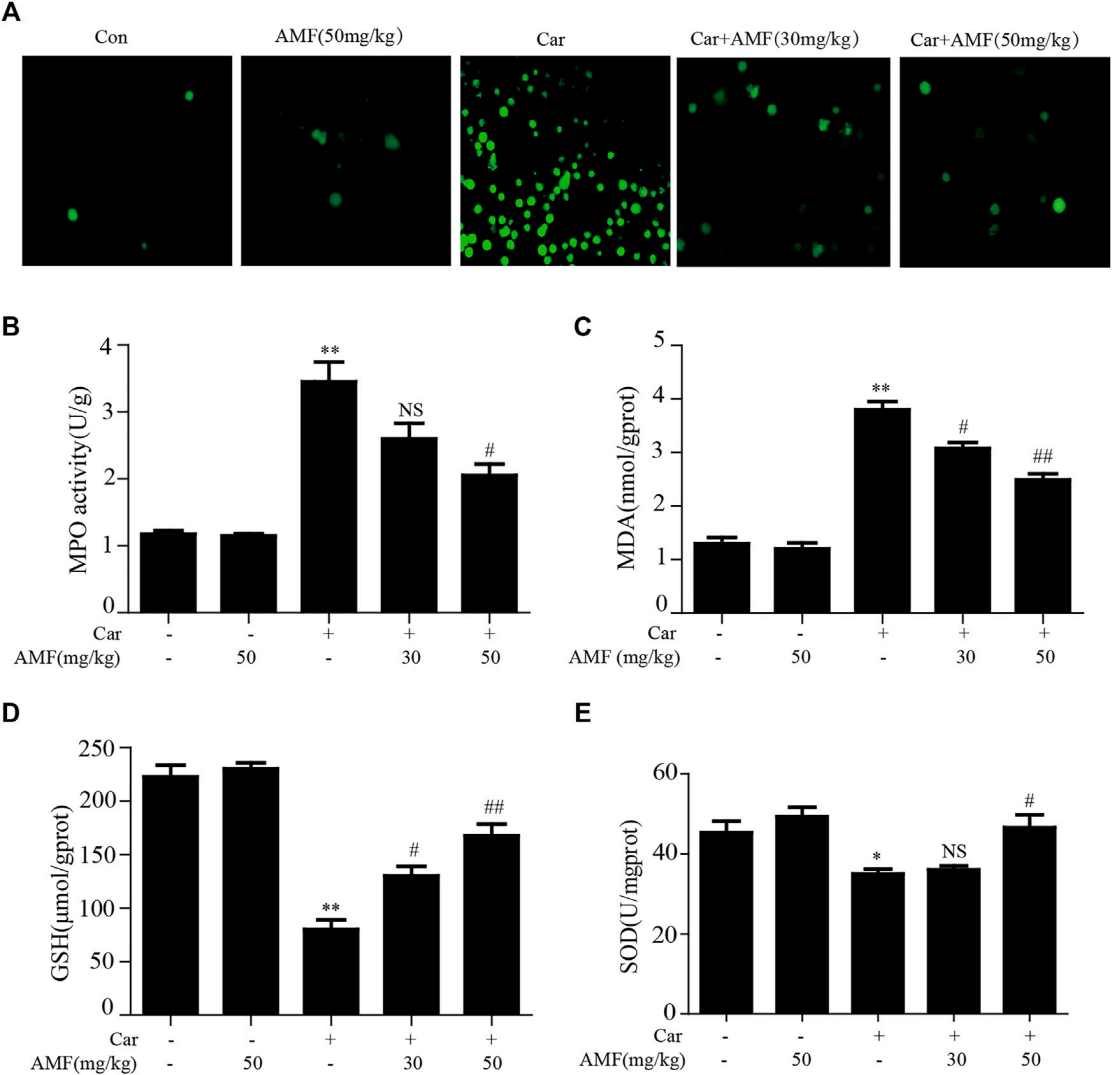
AMF alleviated Car-induced oxidative stress. **(A)** ROS production by leukocytes stained with DCFH-DA was examined with a microscope (original magnification ×200). **(B–E)** The MPO, MDA, GSH, and SOD levels in lung tissues. The data are expressed as the mean ± SEM. *n* = 5 per group. *p < 0.05 and **p < 0.01 Car group vs. control group; #*p* < 0.05, ##*p* < 0.01, and NS *p* > 0.05 Car + AMF group vs. Car group.

As shown in Figure 3A, exposure to Car caused excessive ROS production, while treatment with AMF reduced ROS levels. As shown in Figures 3B,C, the levels of MDA and MPO were sharply increased in the model group compared with the control group, but the increases were markedly inhibited by AMF treatment. In contrast, as shown in Figures 3D,E, the levels of GSH and SOD were significantly decreased by Car but were partially restored by AMF treatment. These results indicated that AMF played an antioxidative role in Car-induced pleurisy and lung injury.

### AMF Exerted an Antioxidative Effect on Car-Induced Lung Injury by Regulating Nrf2 and NADPH Oxidase

**FIGURE 4:**
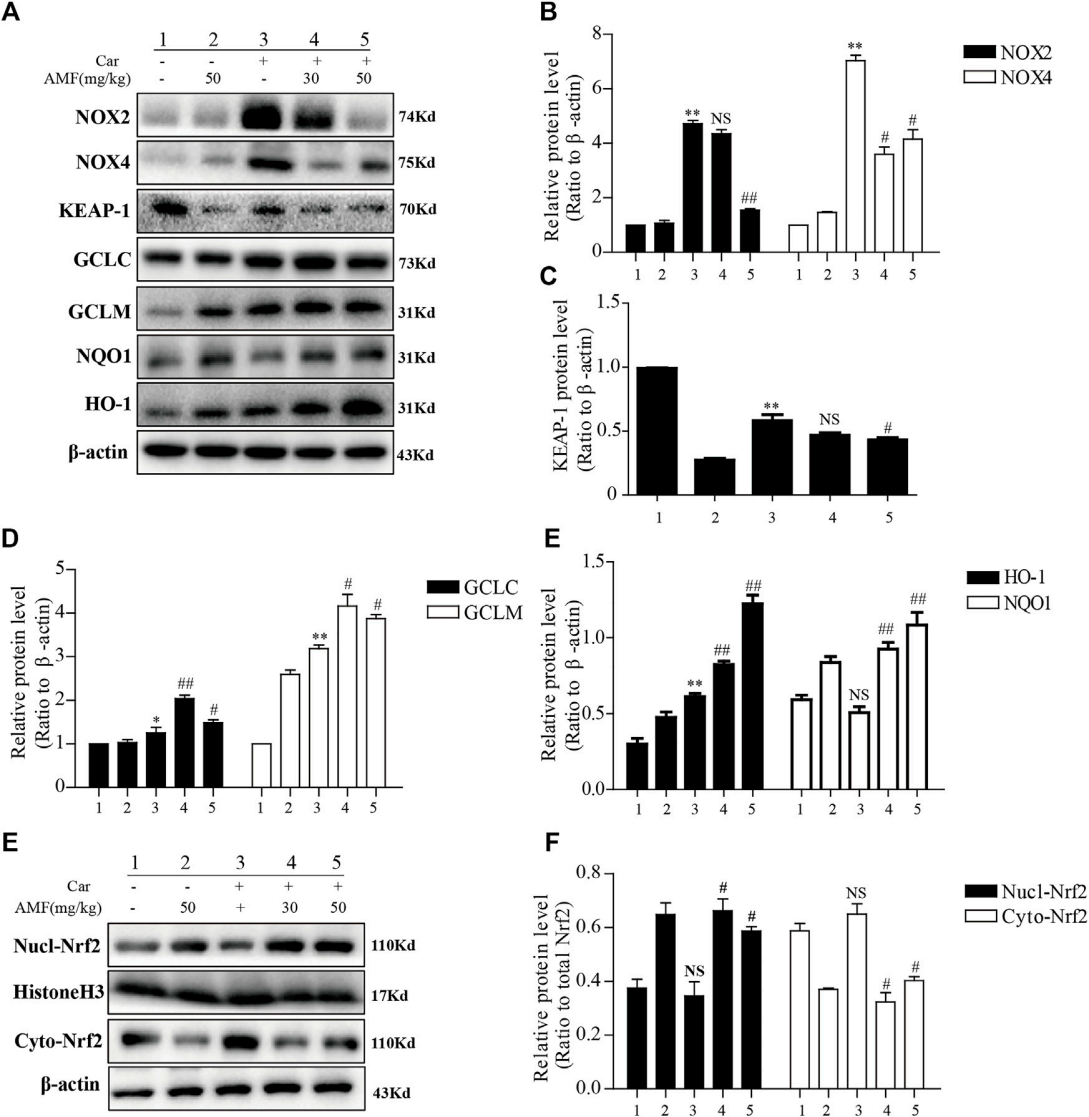
AMF inhibited NOX2 and NOX4 but activated Nrf2 in our pleurisy model. **(A–E)** The expression levels of NOX2, NOX4, and the target proteins of the KEAP1-Nrf2 pathway, including HO-1, NQO1, GCLM, and GCLC. **(E,F)** The levels of Nrf2 in the nucleus and cytoplasm. The data are expressed as the mean ± SEM. *n* = 5 per group. *p < 0.05, **p < 0.01, and NS *p* > 0.05 Car group vs. control group; #*p* < 0.05, ##*p* < 0.01, and NS *p* > 0.05 Car + AMF group vs. Car group.

We examined the expression of certain oxidation-related enzymes by western blotting. Figures 4A–E show that Car induced oxidative stress; correspondingly, NOX2 and NOX4 were upregulated in the Car group compared to the control group. Moreover, the expression of some antioxidative enzymes downstream of the Nrf2 signaling pathway, including GCLC, GCLM, NQO1, and HO-1, was stimulated by Car. AMF promoted the degradation of KEAP-1 (Figures 4A,C), facilitated the nuclear translocation of Nrf2 (Figures 4E,F), and further upregulated the expression of GCL, NQO1, and HO-1, thus enhancing resistance to Car-induced oxidative stress.

### AMF Treatment Blocked the Phosphorylation of NF-κB and Downregulated the Expression of iNOS and COX2

**FIGURE 5:**
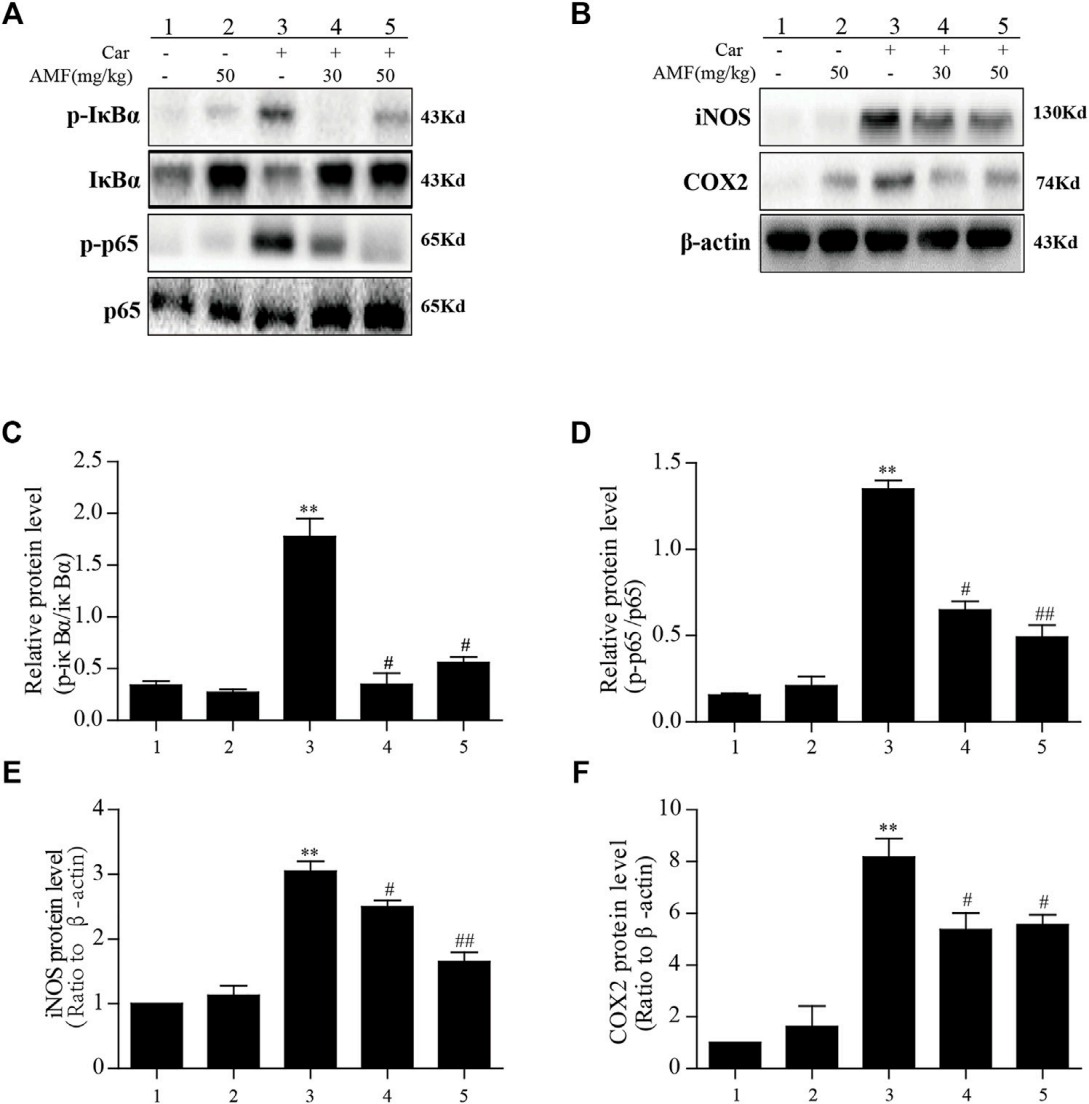
AMF inhibited the activation of NF-κB and increased iNOS and COX2 expression in lung tissue after Car administration. **(A,C,D)** AMF blocked the phosphorylation of IκBα and p65. **(B,E,F)** AMF suppressed the Car-induced increases in the expression of iNOS and COX2. The data are expressed as the mean ± SEM. *n* = 5 per group. *p < 0.05 and **p < 0.01 Car group vs. control group; #*p* < 0.05 and ##*p* < 0.01 Car + AMF group vs. Car group.

The results described above indicated that AMF could relieve the inflammatory response induced by Car; however, further investigation was required to reveal the mechanism. As shown in Figure 5, we found that in the model group, p-p65 and p-IκBα were markedly upregulated in response to Car, whereas in the AMF treatment groups, AMF downregulated the phosphorylation of these signaling pathway factors (Figures 5A,C,D). In addition, Car increased the protein levels of iNOS and COX2 in the mice in the model group. In the AMF treatment groups, the levels of iNOS and COX2 were obviously decreased compared to those in the model group (Figures 5B,E,F). These findings indicated that AMF protected against Car-induced lung injury by downregulating the expression of iNOS and COX2 and the phosphorylation of NF-κB.

### AMF Alleviated the Inflammatory Response by Blocking the ERK, JNK, and STAT3 Signaling Pathways

**FIGURE 6:**
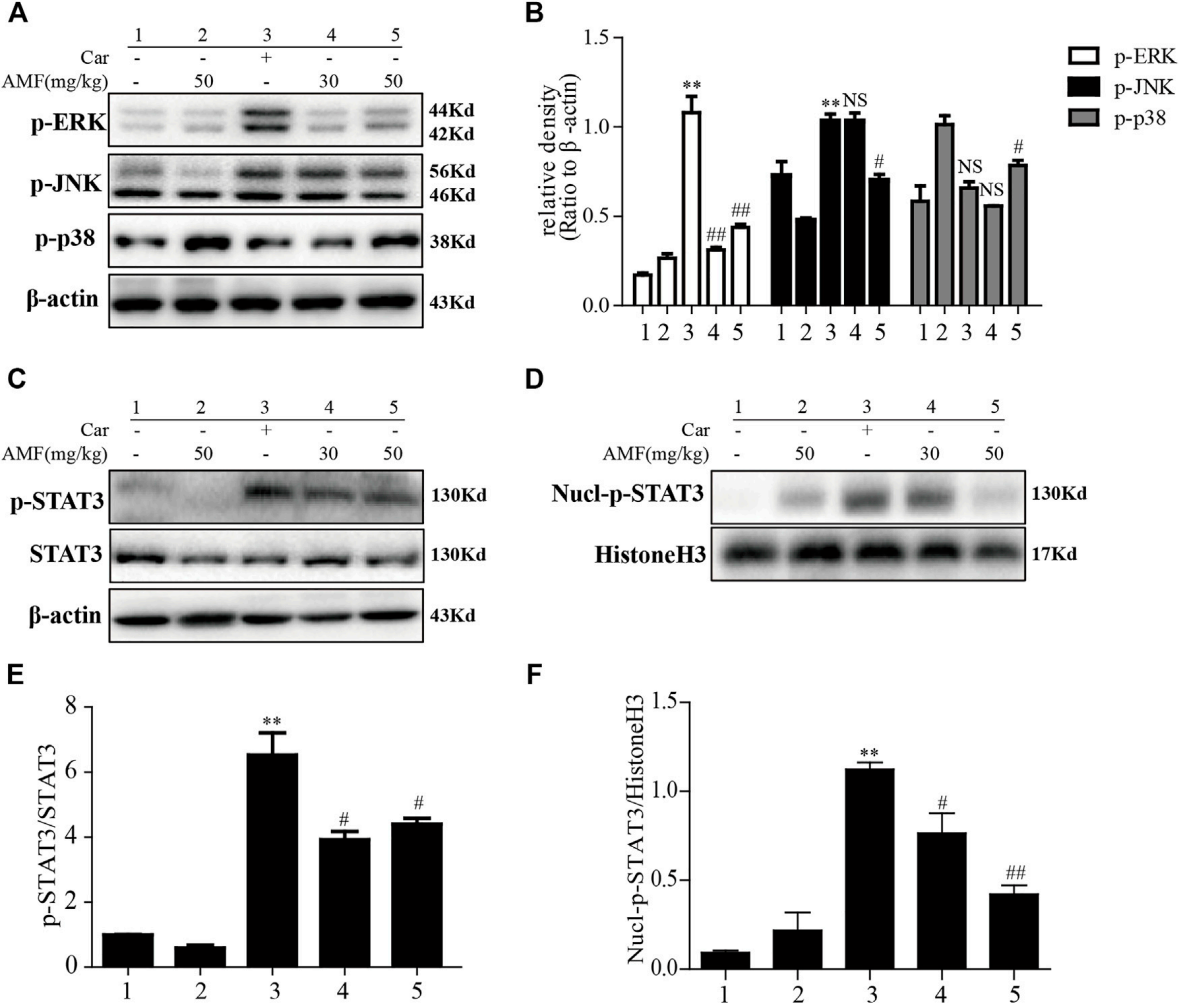
AMF inhibited activation of the MAPK and STAT3 pathways in Car-induced lung injury. **(A,B)** Activation of the MAPK pathway in Car-induced lung injury. **(C,E)** AMF inhibited the Car-induced activation of STAT3. **(D,F)** AMF prevented the nuclear translocation of p-STAT3. The data are expressed as the mean ± SEM. *n* = 5 per group. *p < 0.05 and **p < 0.01 Car group vs. control group; #*p* < 0.05, ##*p* < 0.01, and NS *p* > 0.05 Car + AMF group vs. Car group.

After the injection of Car into the pleural cavity, the phosphorylation of ERK and JNK in the lung tissues was obviously upregulated in the model group. However, treatment with AMF downregulated the phosphorylation of these signaling pathway factors, as shown in Figures 6A,B. Moreover, as shown in Figures 6C–F, we found that Car activated STAT3 and facilitated the nuclear translocation of p-STAT3. AMF treatment not only downregulated the phosphorylation of STAT3 but also blocked the nuclear translocation of p-STAT3.

### AMF Protected Against Car-Induced Pleurisy and Lung Injury via Nrf2 Activation

To investigate whether AMF protects against Car-induced pleurisy and lung injury via the Nrf2 signaling pathway, we examined C57BL/6 Nrf2^-/-^ mice.

**FIGURE 7:**
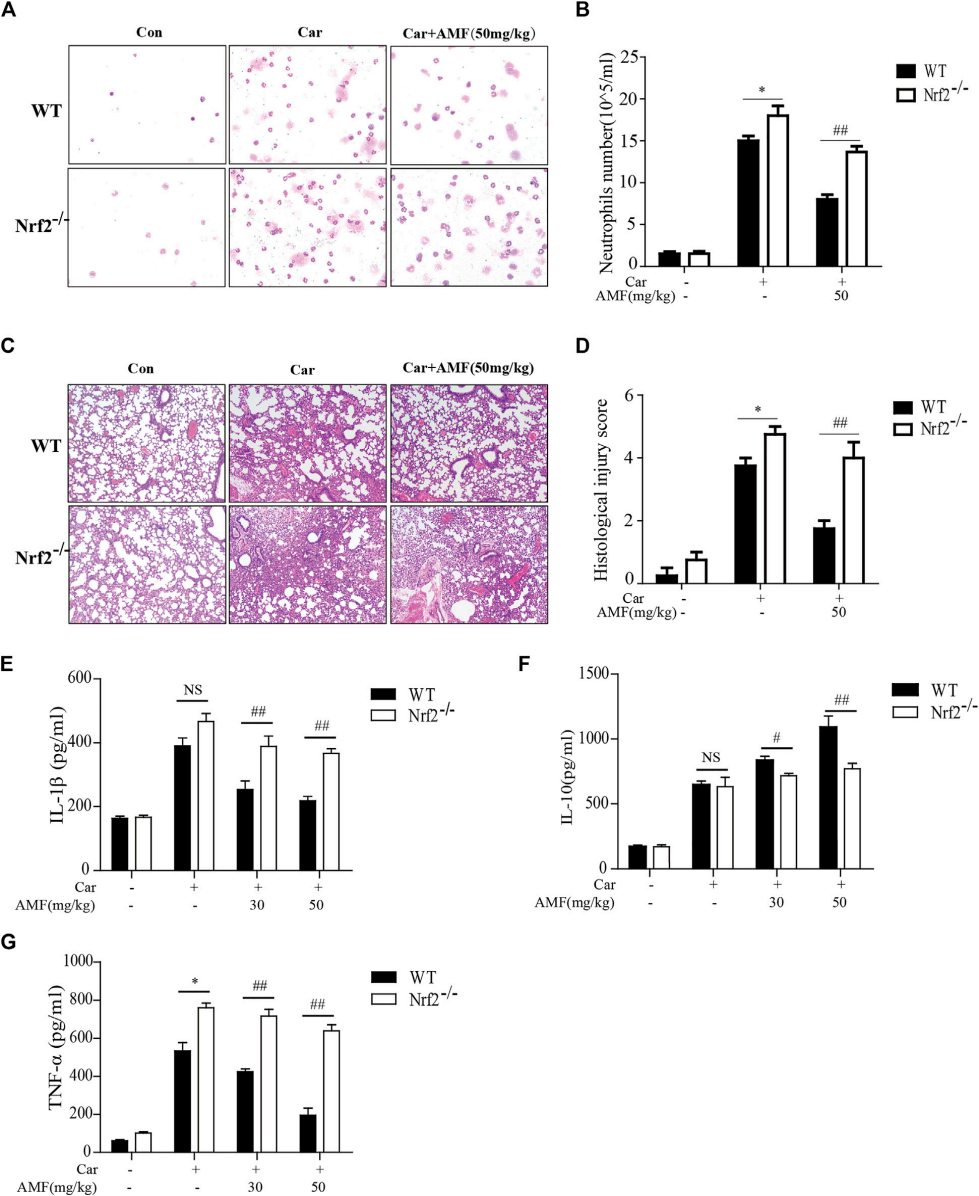
AMF exerted a protective effect against Car-induced pleurisy and lung injury through Nrf2 activation. **(A)** Inflammatory cells were examined by Wright-Giemsa staining (magnification ×400). **(B)** The number of neutrophils in pleural exudate. **(C)** The pathological changes in lung tissues were analyzed by H&E staining (original magnification ×100). **(D)** The histological lung injury score was graded as previously described. **(E–G)** IL-1β, IL-10, and TNF-α levels in exudates were measured by ELISA. The data are expressed as the mean ± SEM. *n* = 5 per group. *p < 0.05, **p < 0.01, and NS *p* > 0.05 WT mice vs. Nrf2^-/-^ mice in the Car group; #*p* < 0.05 and ##*p* < 0.01 WT mice vs. Nrf2^-/-^mice in the Car + AMF group.

As shown in Figures 7A,C, the Wright-Giemsa staining results showed that Car caused massive exudation into the pleural cavity in WT and Nrf2^-/-^ mice. AMF decreased the number of neutrophils to a greater extent in WT mice than in Nrf2^-/-^ mice. Similarly, H&E staining revealed that Car caused lung injury in both WT and Nrf2^-/-^ mice. AMF treatment obviously improved Car-induced lung injury in WT mice; however, this effect was not observed in Nrf2^-/-^ mice (Figures 7B,D). We found that IL-1β and TNF-α levels were obviously increased by Car in both WT and Nrf2^-/-^ mice, while AMF treatment inhibited the expression of these factors in WT mice but not in Nrf2^-/-^ mice. Furthermore, Car increased IL-10 levels in both types of mice; however, AMF treatment further increased the level of IL-10 in WT mice but not in Nrf2^-/-^ mice (Figures 7E–G). Our results indicated that AMF exerted its anti-inflammatory effect via Nrf2.

### AMF Blocked Related Inflammatory Pathways Activated by Car via Nrf2

**FIGURE 8:**
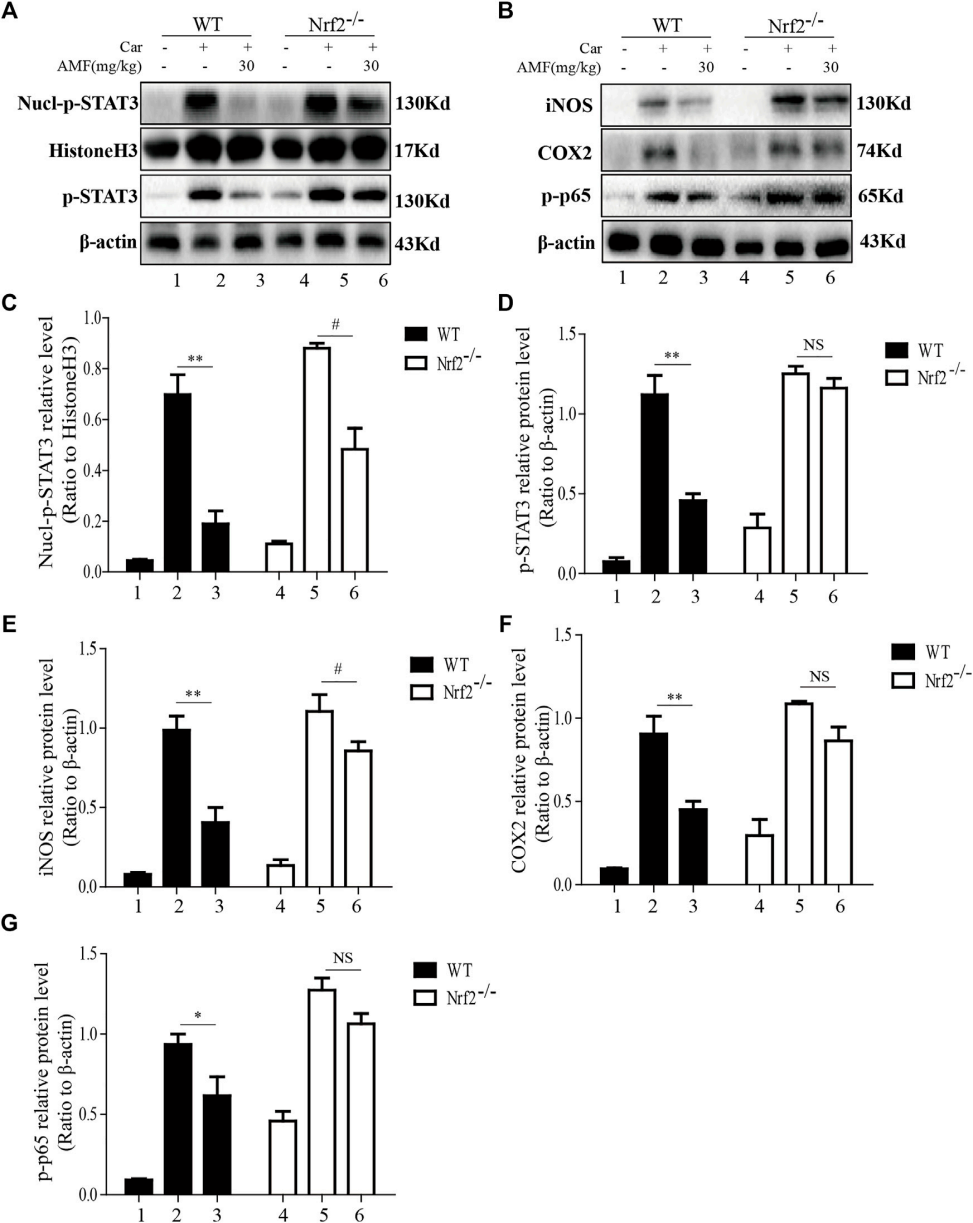
AMF failed to block STAT3 and NF-κB p65 activation in Nrf2^-/-^ mice. **(A,C,D)** The inhibitory effect of AMF on total and nuclear p-STAT3. **(B,E-G)** The expression of iNOS, COX2, and p-p65. The data are expressed as the mean ± SEM. *n* = 5 per group. *p < 0.05 and **p < 0.01 Car + AMF group vs. Car group in WT mice; #*p* < 0.05 and NS *p* > 0.05 Car + AMF group vs. Car group in Nrf2^-/-^ mice.

As shown in Figures 8A,C,D, Car injection activated STAT3 phosphorylation and nuclear translocation in the lungs of both WT and Nrf2^-/-^ mice. AMF treatment inhibited STAT3 activation and nuclear translocation in WT mice, but in Nrf2^-/-^ mice, Car-induced STAT3 activation was not suppressed by AMF, whereas p-STAT3 translocation was inhibited by AMF. In addition, we found that AMF downregulated iNOS and COX2 expression and inhibited the activation of p65, as previously described, in WT mice. In Nrf2^-/-^ mice, AMF failed to downregulate iNOS and COX2 expression and block the phosphorylation of p65, as shown in Figures 8B,E–G.

### AMF Alleviated Car-Induced Oxidative Stress in Lung Tissues in an Nrf2-dependent Manner

**FIGURE 9:**
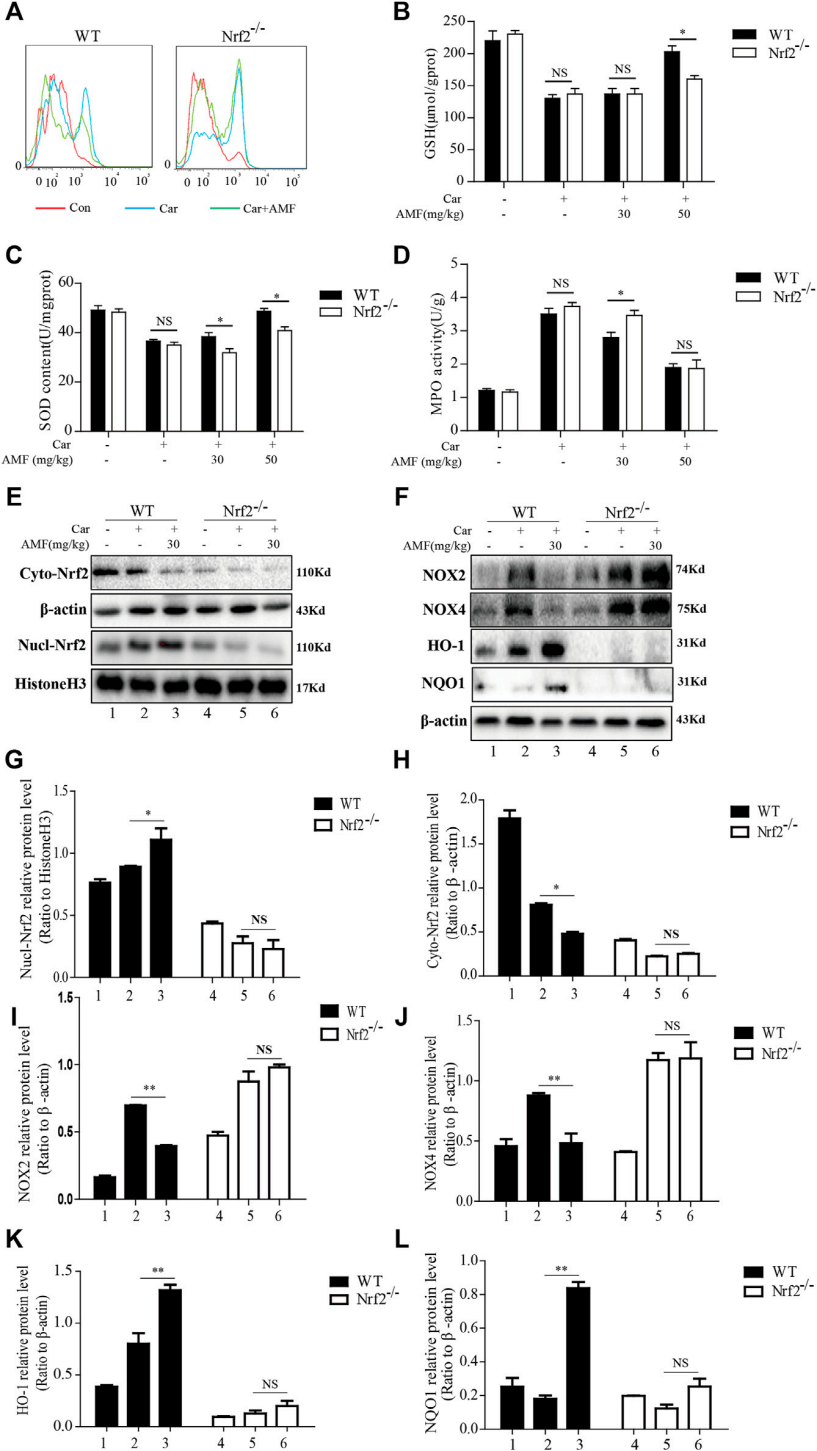
AMF could not alleviate Car-induced oxidative stress in Nrf2^-/-^ mice. **(A)** ROS production by inflammatory cells in the pleural cavity was analyzed by flow cytometry. **(B–D)** GSH, SOD and MPO levels in lung tissues were measured as previously described. **(E,G,H)** The levels of Nrf2 in the nucleus and cytoplasm in WT and Nrf2^-/-^ mice. **(F,I–L)** The expression of NOX2, NOX4, HO-1, and NQO1 in WT and Nrf2^-/-^ mice. The data are expressed as the mean ± SEM. *n* = 5 per group. NS *p* > 0.05, *p < 0.05 in Panel 9B–D. *p < 0.05, **p < 0.01 Car + AMF group vs. Car group in WT mice; NS *p* > 0.05 Car + AMF group vs. Car group in Nrf2^-/-^ mice in Panel 9G–L.

As Nrf2 is pivotal for antioxidation, we next investigated whether AMF protected against Car-induced oxidative stress in an Nrf2-dependent manner. ROS generation was examined by flow cytometry. As shown in Figure 9A, AMF eliminated the excessive ROS induced by Car in WT mice, which was consistent with the ROS immunofluorescence microscopy results (Figure 3A), but this effect was not observed in Nrf2^-/-^ mice. SOD and GSH depletion was inhibited more effectively in WT mice than in Nrf2^-/-^ mice (Figures 9B,C). However, the increase in MPO level induced by Car was abrogated by AMF in both WT and Nrf2^-/-^ mice (Figure 9D). Western blotting results showed that when Nrf2 was absent, AMF failed to downregulate NOX2 and NOX4 expression and failed to activate the expression of downstream genes, as shown in Figures 9E–L.

## Discussion

Oxidative stress and inflammatory responses exert strong effects on the pathogenesis of a Car-induced pleurisy model (Barth et al., 2016). Inflammatory cells release excessive ROS, resulting in exaggerated oxidative damage. In turn, several ROS enhance proinflammatory responses, creating a vicious cycle that exacerbates lung injury (McGarry et al., 2018). Nrf2 is a crucial transcription factor that protects against damage caused by oxidative stress and the inflammatory response (Yamamoto et al., 2018). AMF, a well-known biflavonoid that occurs in many plants, has been shown to exert antioxidative effects via the Nrf2 pathway and to exhibit other beneficial bioactivities and effects, including anti-inflammatory, antidiabetic, and antisenescence effects (Yu et al., 2017). In the present study, we focused on whether AMF, through Nrf2 activation, protects against the pleurisy and lung injury induced by Car.

Diverse pathological processes, such as pleural effusion, inflammatory cell infiltration, interstitial thickening and alveolar hemorrhage, are present in the pathogenesis of Car-induced pleurisy and lung injury, as previously described. We found that AMF alleviated Car-induced increases in inflammatory cell numbers, pleural effusion and pathological changes in lung tissue. Moreover, macrophage activation and neutrophil accumulation result in the production of proinflammatory cytokines, including TNF-α, IL-1β, IL-6, iNOS, and COX2, which are closely associated with the severity of pleurisy and lung injury induced by Car. In contrast, IL-10, an anti-inflammatory factor, suppresses inflammation to protect against various pathological processes (Jung et al., 2017; Mahon et al., 2020). Our results indicated that AMF treatment alleviated F4/80 macrophage infiltration in lung tissue and alleviated TNF-α and IL-1β generation but increased IL-10 production, as indicated by cytokines levels in the supernatant. Taken together, our results indicated that AMF exerted anti-inflammatory effects in Car-induced pleurisy and lung injury.

Car exposure results in oxidative stress via NADPH oxidase activation and inflammatory cell infiltration. The internal antioxidative enzymes SOD and GSH, which are efficient scavengers of deleterious oxygen free radicals, can fight against oxidative stress. In contrast, MPO, which is generated by neutrophil overaccumulation, and MDA, which is produced through lipid peroxidation, facilitate oxidative stress (Barth et al., 2016). Our results indicated that AMF alleviated Car-induced ROS overproduction, MPO/MDA generation and GSH/SOD consumption in lung tissues. Additionally, Nrf2, a central transcription factor associated with oxidative defense, counteracts oxidative stress. In the cytoplasm, Nrf2 binds to KEAP1, which leads to the rapid degradation of Nrf2. However, under exposure to electrophiles or ROS, Nrf2 is released from KEAP1, directly translocates to the nucleus, and then activates the expression of downstream antioxidative genes, including HO-1, NQO1, GCLC, and GCLM (Yamamoto et al., 2018). Our results indicated that AMF not only activated Nrf2 through keap-1 degradation and these downstream antioxidative genes but also downregulated NOX2 and NOX4, suggesting that AMF protects against oxidative stress in Car-induced lung injury.

We further explored the mechanism of the anti-inflammatory effect of AMF in Car-induced pleurisy and lung injury. Accumulating evidence has shown that NF-κB is involved in the Car-induced inflammatory response. In addition, AMF can inhibit the NF-κB pathway to alleviate inflammation (Alam et al., 2020). Similarly, our results indicated that AMF inhibited the Car-induced phosphorylation of IκB and NF-κB p65. Moreover, AMF downregulated the expression of iNOS and COX2. MAPKs are also responsible for Car-induced inflammation. Some reports have suggested that AMF inhibits inflammation and tumor growth by blocking MAPK pathways (Zha et al., 2016). In contrast, AMF has been shown to activate Nrf2 through the ROS-mediated p38-AKT pathway in human keratinocytes (Wahyudi et al., 2018). Interestingly, our results indicated that AMF inhibited Car-induced ERK and JNK activation and that this inhibition was associated with its anti-inflammatory effects. However, AMF activated the p38 MAPK pathway, which might be related to Nrf2 activation. As a member of the STAT family, STAT3 is phosphorylated at serine 727 (S727), which enhances its transcriptional activity and is vital for the production of many proinflammatory cytokines, such as IL-6, IL-1β and COX2 (Hillmer et al., 2016). In addition, many studies have shown that AMF can block STAT3 activation in various cancers (Johnson et al., 2018). Surprisingly, we found that AMF treatment suppressed the Car-induced phosphorylation and nuclear translocation of STAT3. Overall, our study indicated that AMF suppressed the Car-induced inflammatory response by blocking NF-κB, ERK, JNK, and STAT3 activation.

Given that oxidative stress and inflammation are important causes of the pleurisy and lung injury induced by Car and that Nrf2 coordinates oxidative stress and the inflammatory response, we used Nrf2^-/-^ mice to explore whether AMF protects against Car-induced pleurisy and lung injury via Nrf2 activation. As expected, AMF failed to relieve the inflammatory response and pathological changes in lung tissues and the oxidative stress induced by Car in Nrf2^-/-^ mice. Thus, our results indicated that in the presence of Nrf2 deficiency, AMF was not effective against Car-induced pleurisy and lung injury. The STAT3 and NF-κB inflammatory pathways have been suggested to interact with Nrf2. The involvement of Nrf2 in AMPK-driven STAT3 inactivation protects against endotoxic inflammation in the lung (Gong et al., 2020). In addition, a recent report indicated that Nrf2 deficiency exacerbated cardiac damage triggered by Ang II infusion by enhancing STAT3 activation (Chen et al., 2019). Accordingly, our results indicated that AMF failed to inhibit Car-induced STAT3 activation in Nrf2^-/-^ mice. Furthermore, Nrf2 has been demonstrated to inhibit NF-κB itself or downstream genes of NF-κB, which suggests crosstalk between NF-κB and Nrf2 (Sivandzade et al., 2019). In addition, Nrf2 suppression significantly weakens the inhibitory effect of Maresin 1 on NF-κB in DSS-induced colitis (Qiu et al., 2020). Similarly, our results indicated that NF-κB p65 activation and the increased expression of iNOS and COX2 caused by Car were not suppressed by AMF in Nrf2^-/-^ mice.

## Conclusion

In summary, our study suggested that AMF improved Car-induced pleurisy and lung injury by inhibiting the oxidative and inflammatory pathways. In addition, surprisingly, we found that AMF exerted a protective effect by activating Nrf2. This research indicates that AMF has therapeutic potential for inflammatory diseases of the respiratory system.

## Data Availability

The original contributions presented in the study are included in the article/Supplementary Material, further inquiries can be directed to the corresponding authors.
